# A Tailorable and Transferable Flexible Patch for Simultaneous Electrostimulation and Electro‐Controlled Drug Delivery in Wound Management

**DOI:** 10.1002/smsc.202500570

**Published:** 2026-04-21

**Authors:** Rongyan He, Qiuyu Cao, Wenhui Yan, Shuting Xiao, Xiaoying Liang, Yuxiu Ye, Dongting Zhangsun, Sulan Luo

**Affiliations:** ^1^ Guangxi Key Laboratory of Special Biomedicine School of Medicine Guangxi University Nanning China

**Keywords:** electro‐controlled drug delivery, electrostimulation therapy, flexible electronic patch, wound management

## Abstract

Effective wound management is crucial for improved outcomes through timely, personalized therapies. Current dressing‐based treatments lack dynamic regulation and microenvironment modulation, leading to suboptimal healing. Integrating electrical stimulation therapy with electro‐controlled drug delivery promotes skin generation, ensures precise dosing, and accelerates healing. Flexible electronic patches are ideal platforms for these synergistic therapies but face challenges: rigid post‐manufacture shapes and sizes limit adaptability to diverse wound geometries, and substrate materials struggle to meet both wound‐healing and electronic requirements. This study develops a tailorable, transferable flexible electronic patch with a hexagonal modular design, enabling shape/size customization while maintaining electrical continuity. The water‐transfer printing (WTP) technique is used to conveniently transfer conductive chondroitin sulfate (CS) hydrogel (as electrodes and drug reservoirs) and silver circuits onto clinical dressings. This method endows clinical dressings with functionality of electro‐controlled drug release and electrical stimulation, simultaneously addressing substrate performance limitations of flexible electronic patches. The patch demonstrates uniform electrical signal transmission, precise electro‐responsive drug control in CS hydrogels, and adaptability to wounds of different geometries. It retains electrostimulation and electro‐controlled drug delivery performance after cutting, significantly promoting wound healing. This study provides a potential approach to support the advancement of flexible electronics research and clinical translation.

## Graphical abstract

A novel flexible electronic patch integrates electrical stimulation and electro‐controlled drug delivery for advanced wound management. Featuring a modular hexagonal design, the device is tailorable to diverse wound shapes while maintaining electrical continuity. Utilizing water‐transfer printing (WTP), the patch seamlessly integrates with clinical dressings, offering a customizable and effective platform to accelerate tissue regeneration.

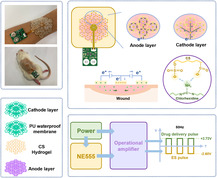

## 1 Introduction

Wound management remains a critical clinical challenge, directly affecting patient recovery outcomes [1]. Effective wound management reduces infection risks, accelerates tissue regeneration, and minimizes scarring, thereby lowering healthcare burdens. Electrostimulation therapy has gained increasing attention for its bio‐regulatory mechanisms [2]. By delivering physiologically relevant electric fields that mimic endogenous bioelectric signals, this approach enhances directional epidermal cell migration, stimulates angiogenesis, and regulates inflammatory responses, establishing a physical therapeutic strategy [3]. Concurrently, electrically responsive drug delivery systems have advanced localized treatment approaches [4]. These technologies employ electricity‐sensitive carriers to achieve spatiotemporally controlled drug release, improving local drug concentration while minimizing systemic toxicity [5]. The integration of electrostimulation therapy with electrically responsive drug delivery functions within a single electronic patch platform offers a therapeutic approach that enhances outcomes by synchronizing electrical modulation of cellular activity and controlled drug administration [6]. This approach offers new technical pathways for managing complex wound‐healing processes, potentially addressing current limitations in conventional wound care through combined physical and pharmacological interventions.

Flexible electronic patches serve as effective platforms for electrostimulation therapy and electrically controlled drug delivery [7]. For instance, Shirzaei Sani et al. developed an integrated wireless bioelectronic flexible patch for wound healing. The system combines electro‐responsive drug delivery (for anti‐inflammatory and antimicrobial therapy) and exogenous electrical stimulation to promote tissue regeneration and accelerate wound healing [6]. Du et al. developed a drug‐loaded, electronically embedded hydrogel patch combining programable drug release with electrostimulation to accelerate wound healing in diabetic rodent models [8]. Zhang et al. developed a wearable self‐powered microneedle system utilizing conductive drugs, which can effectively synchronize antimicrobial delivery with triboelectric‐driven tissue repair [9]. Sun et al. developed a piezoelectric wound dressing equipped with a “Lock‐ON/OFF” mechanism, which enables precise on‐demand release of hydrophilic antibiotics [10]. However, these reported systems exhibit key limitations: Flexible electronic patches maintain fixed shapes and sizes once manufactured, which results in limited adaptability to diverse wound sizes and geometries during personalized therapy. Moreover, dependence on continuous physical motion for power generation restricts their versatility across different patient needs, while reliance on external mechanical stress to trigger drug release may compromise therapeutic efficacy for immobilized patients. Certain homogeneous conductive materials allow dimensional adjustment through mechanical cutting, such as nanocomposite films consisting of graphene oxide (GO) nanosheets incorporated into conductive polymers [11], a flexible alternating current electroluminescent device constructed by sandwiching an emissive layer between conductive hydrogel electrodes [12], and thermogalvanic cell (TGC) dressing composed of Fe^2+^/Fe^3+^ cross‐linked alginate hydrogel reinforced by nanofibers [13]. However, barriers impede clinical adoption: insufficient biocompatibility validation through rigorous clinical studies, which creates substantial translational challenges between bench research and bedside application, coupled with intricate manufacturing protocols that obstruct cost‐effective production. Our research introduces a hexagonal modular design comprising millimeter‐scale interconnectable circuit units. This configuration enables shape customization through peripheral trimming while maintaining operational integrity. The self‐contained hexagonal units preserve electrical continuity post‐modification via optimized current routing, meeting the demand for customizable shapes and dimensions tailored to wound morphology.

Another critical challenge in existing flexible electronic patch development lies in substrate materials. The substrate materials of flexible electronic patches must reconcile multiple functional requirements, including appropriate mechanical strength, rapid exudate absorption, balanced breathability with moisture retention, inherent antimicrobial activity, and good biocompatibility. These functions need to maintain stability during electronic device fabrication and operation, which creates substantial material engineering challenges. Although researchers have developed prototype substrates (e.g., MXene‐based textile [14], gelatin‐based hydrogels [15], bioresorbable metal foils of Mo [16]) for flexible electronic patches, current progress in this field remains preliminary compared to the mature wound dressing industry, where clinically approved products already deliver proven performance. In this study, we address this constraint through water‐transfer printing (WTP) technology. WTP has emerged as a powerful technique for fabricating conformal bioelectronics by decoupling the high‐energy electronic fabrication process from the fragile receiver substrate [17, 18]. The WTP process utilizes a water‐soluble sacrificial carrier, typically polyvinyl alcohol (PVA), which rapidly hydrates and dissolves upon contact with water. This dissolution leaves an ultrathin, substrate‐free electronic pattern supported entirely by fluid surface tension [19]. Because these electronics possess negligible bending stiffness in their floating state, they can be seamlessly transferred onto complex, micro‐textured geometries via uniform hydrostatic pressure during immersion. This fluid‐driven mechanism circumvents the localized stress concentrations and mechanical mismatches inherent to traditional elastomeric stamping or semi‐rigid flexible substrates [20, 21]. Meanwhile, PVA‐based WTP paper also exhibits good biocompatibility. PVA is an FDA‐approved biomedical polymer widely utilized in clinical wound care, tissue engineering scaffolds, and implantable devices, owing to its superior hydrophilicity, biocompatibility, and noncytotoxic profile. Extensive prior work has confirmed that PVA‐based films and hydrogels exert no adverse effects on the adhesion and proliferation of a broad range of mammalian cells (including fibroblasts, osteoblasts, and mesenchymal stem cells), with excellent in vitro and in vivo biosafety repeatedly verified [22, 23]. Therefore, WTP offers an ideal strategy for the convenient transfer of electronic circuits and conductive hydrogel components to commercially available or preclinical wound dressings, maintaining the structural integrity of the electronic components and hydrogel assemblies, and requiring minimal technical training. By integrating electronic functionality into widely used clinical‐grade or advanced preclinical wound dressings and using them as materials for flexible electronic patches, this strategy facilitates the clinical application of flexible electronics, thereby improving wound management. By incorporating electronic functionalities into widely used clinical‐grade or advanced preclinical wound dressings and using them as substrate materials for flexible electronic patches, this strategy facilitates the clinical translation of flexible electronic technologies for improved wound management.

In this study, we developed a tailorable and transferable flexible electronic patch with dual‐functional integration for simultaneous electrical stimulation therapy and electro‐controlled drug delivery for wound management. The flexible electronic patch demonstrates customizable adaptability to various wound dimensions and geometries through tailoring while maintaining stable electrical performance. Using the WTP technique, we successfully integrated the electronic components with clinically established wound dressing substrates. The patch's tailorable characteristics and substrate transferable capability were characterized through in vitro experiments. We also used rat models with geometrically diverse wounds to evaluate the therapeutic performance of this flexible electronic patch in both electrical stimulation and spatiotemporal control of pharmacological administration. This study presents meaningful progress in application of flexible electronics, potentially enabling personalized wound care solutions through its adaptability to diverse wound types and therapeutic wound dressings.

## 2 Results and Discussion

### 2.1 The Characterization of Chondroitin Sulfate (CS) Hydrogels

**FIGURE 1:**
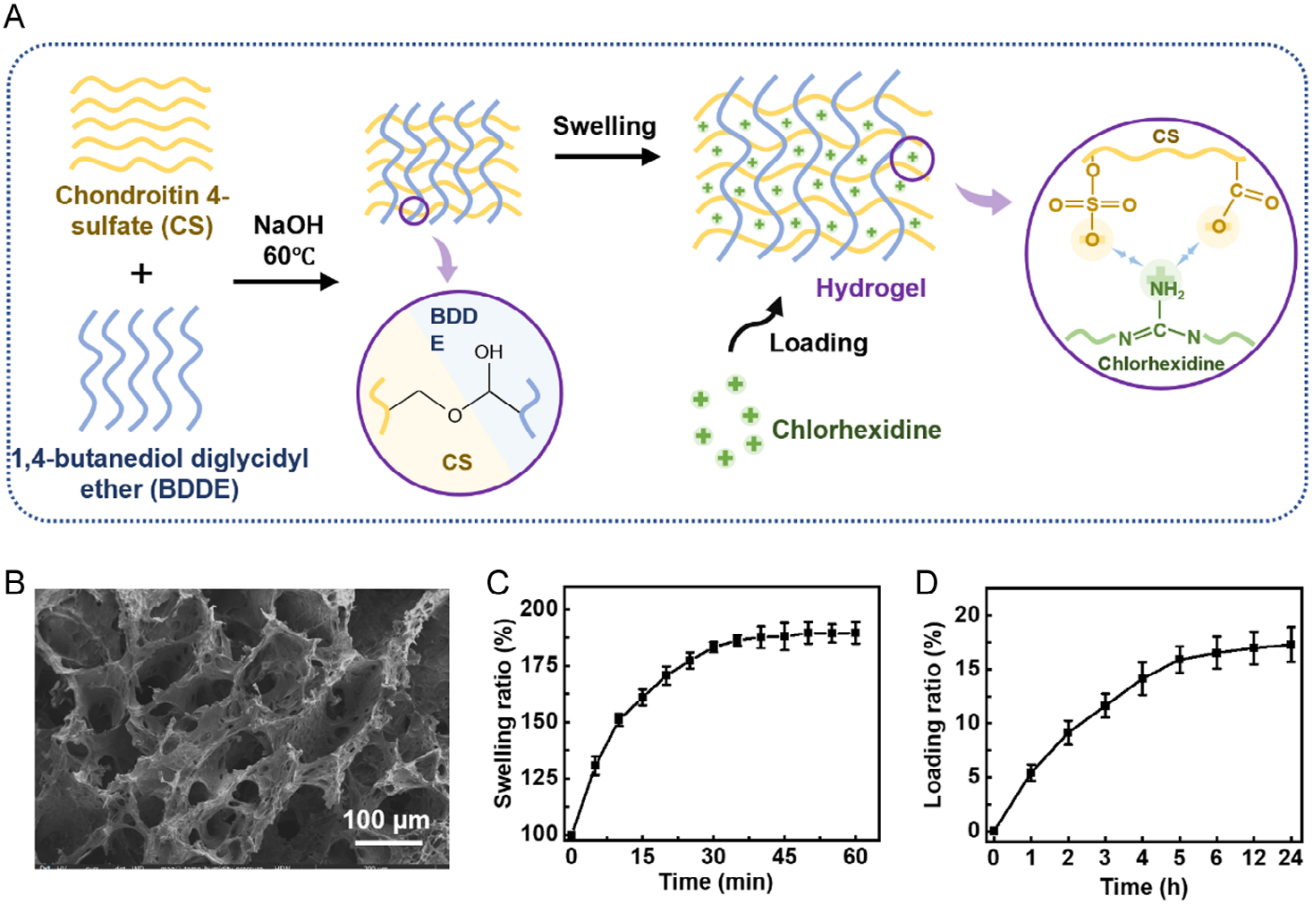
Characterization of CS hydrogels. (A) Schematic illustration of the preparation and drug‐loading process of the CS hydrogel; (B) The SEM image of CS hydrogel; (C) The swelling ratio of CS hydrogel; (D) Drug‐loading ratio of CS hydrogel. The data are presented as mean ± SD, *n* = 3.

CS is a sulfated glycosaminoglycan abundantly present in the extracellular matrix of human tissues, exhibiting superior biocompatibility and carrying a negative charge due to its rich content of sulfate groups and carboxylic acid groups [24], which can be used for dual applications as electrical stimulation therapy electrodes and electro‐controlled drug reservoirs. At the same time, CS demonstrates anti‐inflammatory functions and regulates cellular activities, such as migration and receptor binding, showing remarkable wound‐healing capacity and bioactivity at the cellular level [25]. CS promotes re‐epithelialization and prevents scar formation and has been FDA‐approved for burn wound treatment [26]. The CS hydrogel network contains abundant negatively charged sulfate and carboxylate groups, which enable highly efficient electrostatic loading of the positively charged chlorhexidine. The CS hydrogel was prepared by heating a mixture of sodium chondroitin sulfate A and 1,4‐butanediol diglycidyl ether under alkaline conditions for crosslinking (Figure 1A, Figure S1). Scanning electron microscopy (SEM) revealed a loosely porous three‐dimensional network structure (Figure 1B). Swelling tests showed that CS hydrogel is stable in aqueous environments and can rapidly swell (183% within 30 min), followed by gradual swelling (190% at 60 min) (Figure 1C). In this work, we select chlorhexidine as the therapeutic agent due to its broad‐spectrum antimicrobial properties, which are essential for managing wound bioburden and preventing infection‐related healing delays [27, 28, 29]. In addition, the negatively charged sulfate and carboxylate groups in CS hydrogel enable electrostatic loading of positively charged chlorhexidine. To evaluate drug‐loading capacity, CS hydrogels were immersed in 0.1 wt% chlorhexidine acetate solution. A 17% loading rate was achieved after 12 h (Figure 1D). To confirm the cationic drug affinity of CS hydrogel, we used methylene blue and cationic brilliant red 4G as model cationic drugs, while methyl orange, brilliant blue, carmine, and direct blue 15 as model anionic drugs. We observed that cationic drugs progressively entered hydrogels, while anionic drugs exhibited negligible loading (Figure S2). Since wound infection significantly impedes healing, the high‐capacity loading of chlorhexidine enabled by the CS hydrogel's electrostatic properties serves to endow the dressing with the essential antibacterial activity required for an ideal wound management platform. To explore this capability, we placed the hydrogels in the center of culture media inoculated with *Escherichia coli* (*E. coli*) and *Staphylococcus aureus* (*S. aureus*). After 24 hr of incubation, the hydrogels exhibited antibacterial activity against both strains, with a more pronounced effect against *E. coli*. The inhibition zone area of *E. coli* and *S. aureus* was 2.51 ± 0.24 cm^2^ and 1.56 ± 0.10 cm^2^ (Figure S3).

### 2.2 The Fabrication of Flexible Electronic Patch

**FIGURE 2:**
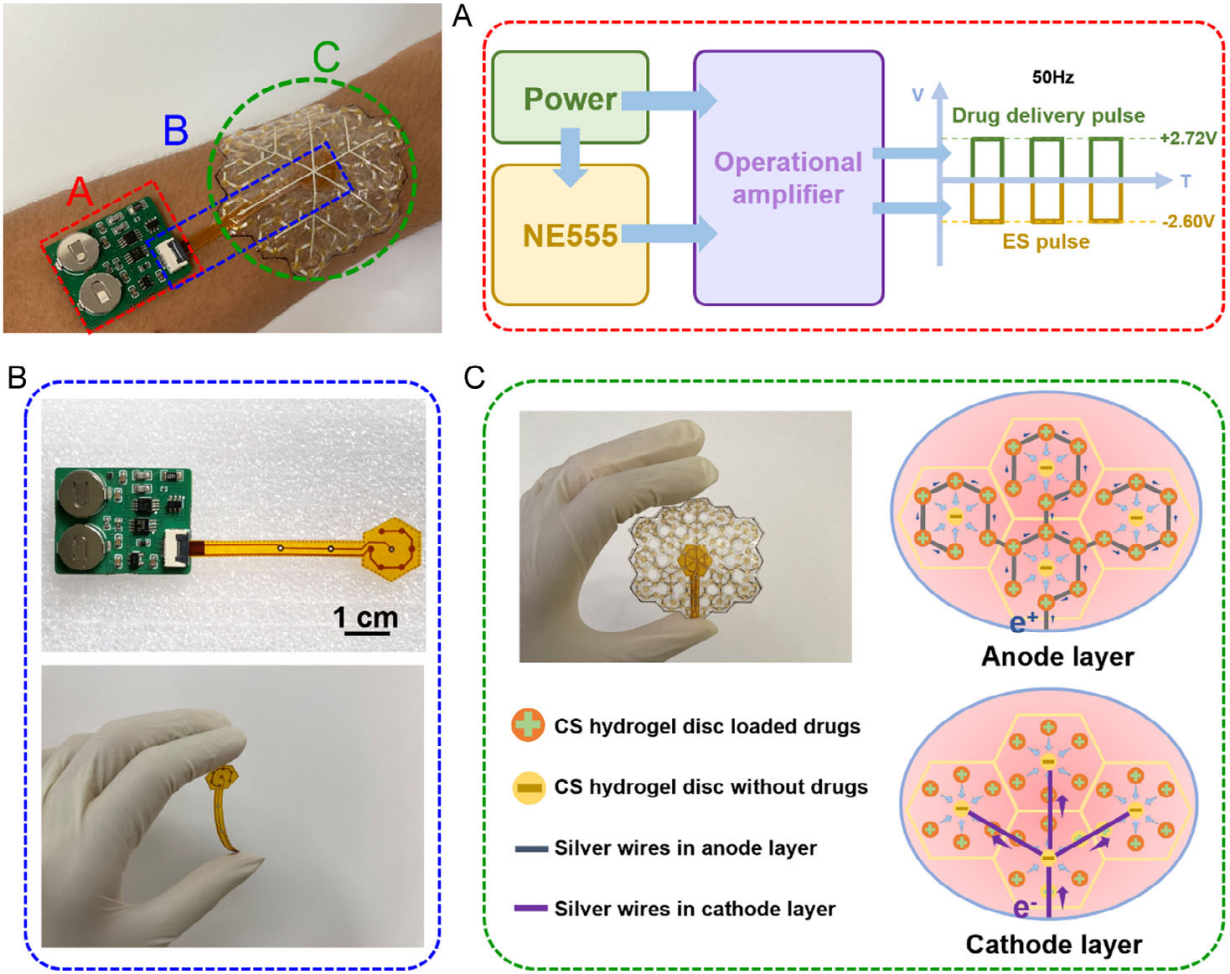
Photograph of wearable device for wound management. It consists of three components: (A) A miniaturized circuit board for generating square‐wave signals; (B) FPC for powering the flexible electronic patch; (C) The flexible electronic patch.

**FIGURE 3:**
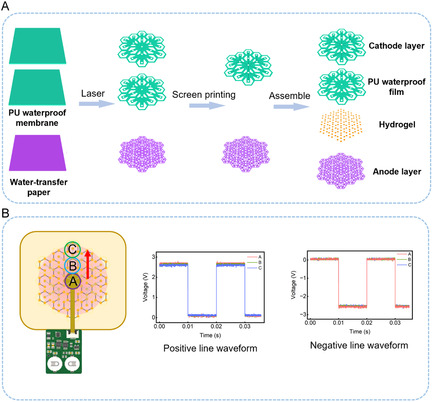
The fabrication process and voltage waveforms of the flexible electronic patch. (A) The fabrication process of the flexible electronic patch; (B) The voltage waveforms of the central hexagonal unit and the peripheral hexagonal units: The central hexagonal unit; The next level of hexagonal units; The outermost hexagonal units. The data are presented as mean ± SD, *n* = 3.

The wearable wound management device consists of three components: a miniaturized control circuit for generating square‐wave signals (A), flexible printed circuit (FPC) for powering the flexible electronic patch (B), and the flexible electronic patch (C), which enables electro‐controlled drug release and electrostimulation therapy. The CS hydrogels are used as the functional core of flexible electronic patches (C). By connecting to positive and negative electrode wires, these hydrogels have a dual function: both for electrical stimulation and for releasing drugs under the control of square waves (Figure 2). In recent studies, square waves are often used as the driving force for electrical stimulation [6, 30]. Compared to continuous direct current and sine waves, square‐wave pulsed stimulation allows for capacitive discharge of tissue at lower levels, effectively mitigating harmful pH changes, preventing thermal damage, and eliminating the risk of electrochemical burns, while still providing the high‐intensity directional polarity required for accelerated tissue regeneration [3, 31]. The circuit design outputs a square‐wave signal with a frequency of 50 Hz, a 50% duty cycle, and peak voltage of + 2.72 V and −2.60 V through a dual‐channel amplifier, where one channel functions as a voltage follower to deliver a positive square‐wave signal for electro‐controlled drug release, while the other channel acts as an inverter to produce a negative square‐wave signal for electrostimulation for wound healing (Figure 2A and Figure S4). The FPC connects to the central smallest unit of the flexible electronic patch via an external power source, ensuring power supply to the patch (Figure 2B). The flexible electronic patch is constructed by interconnecting the smallest hexagonal units with electrical stimulation and electro‐controlled drug release functionalities, which expand outward. Trimming of the patch to desired shapes and sizes is achievable as long as the central power‐connected smallest unit remains intact during cutting. Each hexagonal unit contains seven conductive hydrogel discs: six hydrogel discs connected to the positive electrode for drug release are positioned near the periphery, while one hydrogel disc connected to the negative electrode for electrostimulation therapy is placed at the center. The hydrogel discs in each hexagonal unit are connected in series via silver wires, and the central hexagonal unit is connected in series to the outer hexagons in the next level of expansion (Figure S5). Silver‐based materials have a long‐standing track record of clinical use as antibacterial agents in wound care products, with their antibacterial efficacy and cytocompatibility at clinically relevant safe doses extensively validated [2, 32, 33]. Hexagonal units at the same level are connected in parallel, with the current direction as shown in Figure 2C. The silver wires are fabricated on different planes using screen printing to prevent crossover of positive and negative electrode wires: silver wires printed on WTP paper connect to the positive electrode, whereas those on PU (Polyurethane) waterproof film connect to the negative electrode, ensuring each wire only contacts the corresponding hydrogel. The CS hydrogels were fabricated into circular discs with diameters of 2 mm and thickness of 1 mm, and embedded within screen‐printed silver conductive tracks, forming a flexible electronic patch (Figure 3A and Figure S6).

### 2.3 The Uniformity of Electrical Signal Transmission in the Flexible Electronic Patch

To verify the electrical signal transmission uniformity within this structural design, we used an oscilloscope to detect the voltage waveforms of the central and peripheral hexagonal units. As shown in Figure 3B, the central hexagonal unit outputs actual positive and negative voltage pulse peaks of + 2.72 V and −2.60 V, respectively. As the 50 Hz pulse waveforms propagate outward to the next level and outermost hexagonal units, the signal experiences only highly marginal attenuation, maintaining consistent peak voltages of + 2.64 V and −2.56 V. This stable electrical transmission guarantees that physiological and pharmacological dosing remains therapeutic across the entire wound bed, regardless of the patch's final customized shape.

### 2.4 The Electro‐Controlled Drug Delivery of Flexible Electronic Patch

**FIGURE 4:**
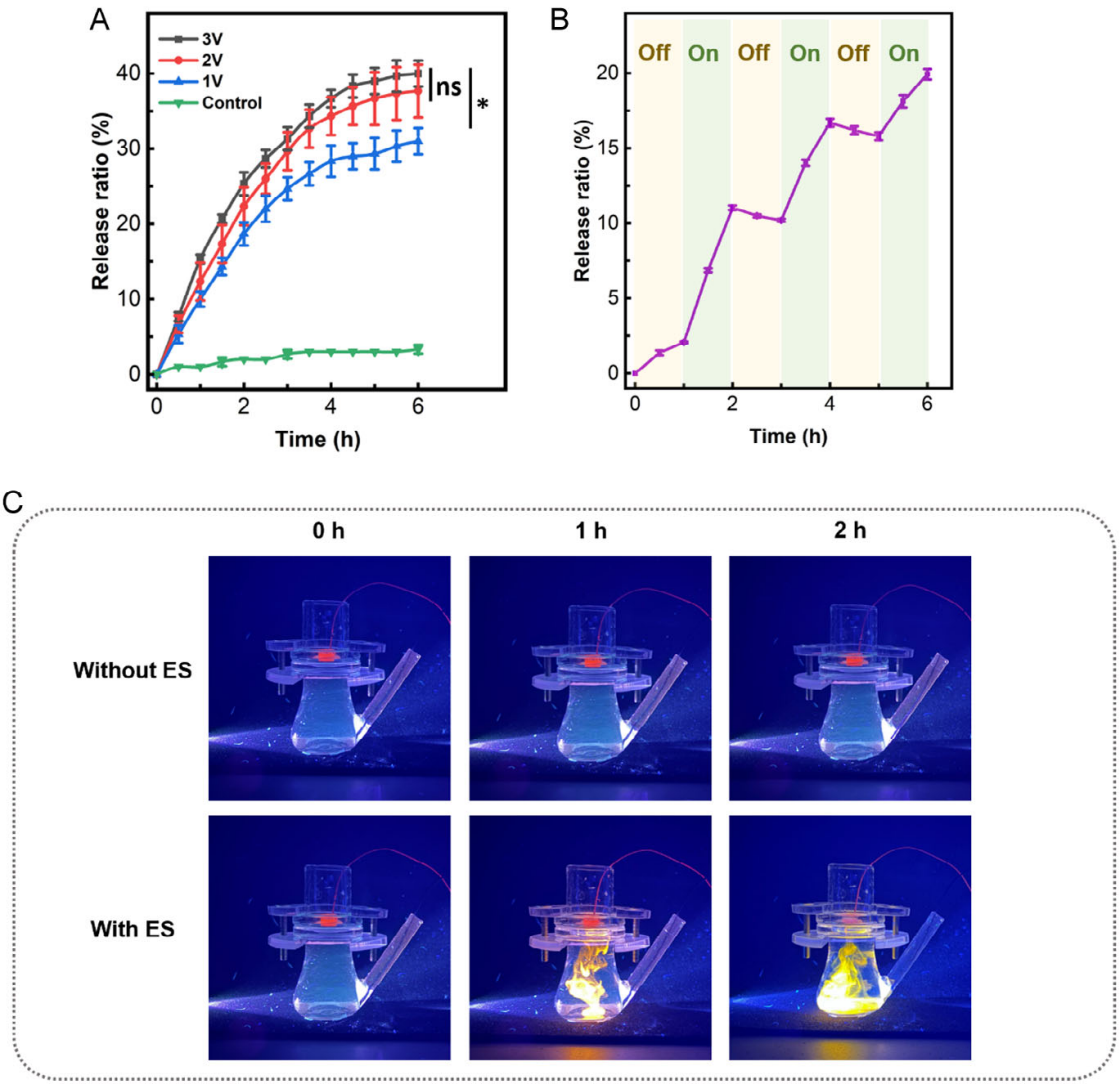
The electro‐controlled drug delivery of flexible electronic patch. (A) Drug release ratio from the CS hydrogel at applied voltages of 0 V, 1 V, 2 V, and 3 V. ns: no statistically significant difference; (B) Drug release ratio during ON phases (1–2 h, 3–4 h, 5–6 hr, at applied voltage of 2 V) and OFF phases (2–3 h, 4–5 h, without applying voltage); (C) The visualization of RhB release. The data are presented as mean ± SD, *n* = 3. ns represents *p* > 0.05, * represents *p* < 0.05.

The flexible electronic patches were subjected to 1V, 2V, and 3V square‐wave voltages (50 Hz frequency), and patches without voltage application were used as control. From Figure 4A, we can see that drug release rates were 3.3 ± 0.6% (control) versus 31.0 ± 1.7% (1V), representing a 10‐fold enhancement. Higher voltages (2V: 37.7 ± 3.5%; 3V: 40.0 ± 1.7%) showed no statistically significant difference. Next, we used intermittent voltage to control drug release. Figure 4B showed that voltage application (2V) induced rapid drug release during ON phases (1–2 h, 3–4 h, 5–6 h), while negligible release occurred during OFF phases (2–3 h, 4–5 h), with partial drug reabsorption via hydrogel swelling. These results demonstrate that the flexible electronic patch enables precise electro‐responsive control of drug release in CS hydrogels. To visually demonstrate the drug release process of CS hydrogel, we used the cationic fluorescent dye Rhodamine B (RhB) as a simulated drug in the drug release experiment. We observed that a significant amount of RhB was released in the experimental group with applied voltage, whereas only negligible RhB release was detected in the control group without applied voltage (Figure 4C and Figure S7).

Electro‐controlled drug release is governed by the interaction between the applied electric field and the CS hydrogel's intrinsic properties. The CS polymer backbone possesses a high density of negatively charged sulfate and carboxyl groups, which enables the high‐capacity electrostatic loading of the cationic drug and provides the internal repulsion necessary to maintain the hydrogel's swollen state. Furthermore, the hydrogel's loosely porous architecture allows the conductive solution to thoroughly permeate the matrix, ensuring uniform propagation of the electric field [34, 35]. When a positive voltage is applied, localized anodic water electrolysis generates a high concentration of hydrogen ions. This protonates the functional groups within the CS matrix, neutralizing their charge and severing the electrostatic bonds anchoring the drug. The freed cationic drug is subsequently expelled from the matrix via electrophoretic repulsion and simultaneous hydrogel deswelling. Upon voltage removal, the localized protons dissipate, allowing the sulfate and carboxyl groups to rapidly re‐ionize. This restores the matrix's high negative charge density and internal electrostatic repulsion, effectively halting the drug release [35, 36, 37].

Meanwhile, it is important to note that the drug delivery capability of this system is governed by the electrostatic properties of the substrate. While this facilitates the high‐capacity loading of cationic drugs, it serves as a functional limitation that restricts the system's applicability for anionic therapeutic agents, which exhibit negligible loading affinity.

### 2.5 Water‐Transfer Performance of the Flexible Electronic Patch

**FIGURE 5:**
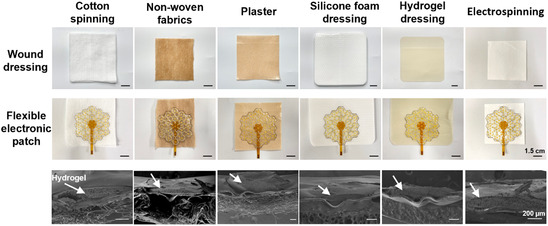
Water‐transfer performance of the flexible electronic patch. The white arrows indicate the CS hydrogels in the SEM images.

Numerous high‐performance wound dressings are commercially available, including cotton spinning, nonwoven fabrics, plaster, silicone foam dressing, hydrogel dressing, and electrospinning dressing (Polyacrylonitrile) (the top row images of Figure 5). These materials typically exhibit excellent biocompatibility, appropriate breathability and moisture permeability, rapidly absorb wound exudate, and prevent bacterial infection, thereby facilitating wound healing, achieving widespread clinical application. We used WTP method to integrate flexible electronic patches onto these wound dressings (the central row images of Figure 5). In our WTP process, screen‐printed silver wires and conductive CS hydrogel discs predeposited on a PVA‐coated polyethylene film are released upon water hydration to form a floating pattern. This pattern is then brought into contact with micro‐textured wound dressings, where subsequent moisture removal permanently fixes the functional layer via van der Waals forces. The transfer process demonstrated remarkable simplicity, enabling medical staff or trained patients to rapidly transfer flexible electronic patches onto chosen substrates (Video 1) for combined electrostimulation and electro‐controlled drug release. The WTP technique enables the use of high‐performance wound dressings as substrate materials for flexible electronic patches. At the same time, it allows commercial dressings to rapidly acquire electrical stimulation capabilities and electro‐controlled drug delivery functions, thereby facilitating the clinical translation of flexible electronic patches.

Our flexible patch adopts a multiplanar assembly architecture, where the screen‐printed positive and negative leads distributed on different planes are inherently protected against short circuits. The matched commercial wound dressing only serves as mechanical support and fulfills basic wound management functions, without impairing the functional properties of the leads under ideal transfer conditions. However, it is difficult to ensure an absolutely flat surface of commercial wound dressings in practical application, and uneven water distribution on the WTP paper during the water‐transfer process will affect the success rate of WTP. These two key factors may interfere with the structural stability of the multiplanar architecture, compromise the preset insulation strategy, and further impair the electrical performance of this flexible patch. Therefore, it is critical to systematically investigate the WTP success rate of the patch on different commercial dressings and rigorously verify the integrity of the insulation strategy and electrical performance of the patch after the water‐transfer process. Herein, we performed comprehensive evaluation experiments targeting the above core indicators for the six types of wound dressings involved in this study.

First, we evaluated the WTP success rate across the six dressings. As shown in Figure S8A, the transfer success rates were 96.15 ± 1.81% for cotton spinning, 96.14 ± 0.80% for nonwoven fabrics, 96.60 ± 1.25% for plaster, 97.71 ± 1.25% for silicone foam dressing, 96.66 ± 1.56% for hydrogel dressing, and 94.20 ± 1.09% for electrospinning dressing, respectively. All dressings achieved a transfer success rate above 94%, with the silicone foam dressing exhibiting the highest value and the electrospinning dressing the lowest. These results confirm that the proposed flexible patch can be successfully transferred onto all six types of dressings with high process stability. To directly validate the integrity of both the interlayer insulation structure and the circuit path after water transfer, we performed SEM characterization. The SEM image (the bottom row images of Figure 5) clearly shows that the distinct boundary between each functional layer is well‐defined and remains fully intact after the water‐transfer process, verifying the stability of the multiplanar insulation strategy. Meanwhile, the SEM image (Figure S8B) demonstrates that the screen‐printed silver wires remain fully intact and continuous following water transfer, with no observable microcracks, fractures, or delamination. These results collectively confirm that neither the interlayer insulating architecture nor the physical structure of the circuit is compromised by the transfer process onto nonconductive wound dressing substrates. To further visually verify the electrical integrity and functional performance of the patch after WTP, LEDs were integrated between the positive and negative electrodes of the outermost hexagonal units; the stable and continuous illumination of LEDs after transfer confirmed that the transfer process did not impair the electrical functionality of the patch (Figure S8C). Overall, the results comprehensively demonstrate that our flexible patch maintains excellent structural stability, intact insulation performance, and reliable electrical functionality after water transfer onto commercial wound dressings, with negligible adverse impact on its performance.

An ideal wound dressing should maintain a moist wound environment and enable efficient drainage of wound exudate. Therefore, characterization of the water transmission rate (WTR) and water vapor transmission rate (WVTR) is particularly critical. Following the transfer of the flexible patch onto the six types of wound dressings, we measured the WTR and WVTR. As presented in Figure S9A, the WTR (unit: kg/m^2^/h) was 17.88 ± 1.29 for cotton spinning, 19.61 ± 2.17 for nonwoven fabrics, 8.74 ± 1.93 for plaster, 13.91 ± 0.65 for silicone foam dressing, 2.47 ± 0.54 for hydrogel dressing, and 18.09 ± 1.30 for electrospinning dressing. Furthermore, as illustrated in Figure S9B, the WVTR (unit: kg/m^2^/day) was 2.17 ± 0.18 for cotton spinning, 2.14 ± 0.25 for nonwoven fabrics, 1.34 ± 0.10 for plaster, 1.57 ± 0.08 for silicone foam dressing, 0.12 ± 0.07 for hydrogel dressing, and 1.95 ± 0.12 for electrospinning dressing.

Notably, the hydrogel dressing presented remarkably low WTR and WVTR values, indicating poor water permeability and breathability. As such, this dressing is well‐suited for application scenarios that require low moisture and gas transmission, such as relatively dry wounds with minimal exudate. In contrast, the other five dressings exhibited significantly higher WTR and WVTR, making them more suitable for wound environments that demand efficient water permeability and breathability.

Given the irreversible process of the WTP, and the gradual dissipation of water and drugs from the hydrogel during treatment, the flexible patch employed in this study is intended for single‐use applications only.

### 2.6 Evaluation of the Therapeutic Efficacy of the Flexible Electronic Patch for Wound Healing In Vivo

**FIGURE 6:**
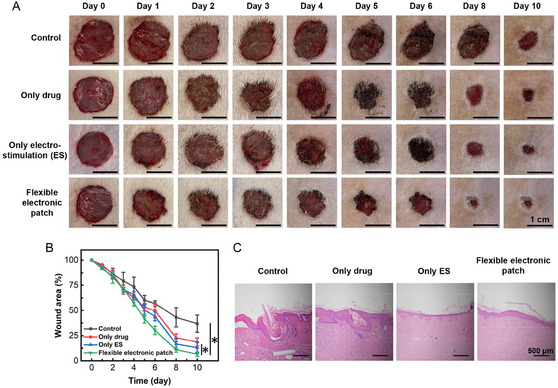
The therapeutic efficacy of the flexible electronic patch for wound healing in vivo. (A) Representative images of wounds; (B) The remaining wound area percentage; (C) H&E‐stained images of wound tissue sections. The data are presented as mean ± SD, *n* = 6. * represents *p* < 0.05.

To evaluate the therapeutic efficacy of the flexible electronic patch for wound healing in vivo, we established rat skin injury models. Rats were divided into four groups: control group (no treatment), only drug group (hydrogel discs loaded with drugs and connected to the positive electrode for drug release), only ES group (electrical stimulation‐only; hydrogel discs without drugs, connected to the negative electrode for electrical stimulation therapy), and flexible electronic patch treatment group (hydrogel discs loaded with drugs and connected to both positive and negative electrodes) (Figure 6, Video 2). On Day 0, circular wounds ≈ 1.5 cm in diameter were created on the same dorsal region of each rat. Over time, wounds in all groups gradually healed, with the flexible electronic patch group exhibiting the fastest healing rate, followed by the ES group, then the drug group, and finally the control group showing the slowest healing (Figure 6A). Statistical analysis of wound area revealed that on Day 6, the remaining wound area was 30.2 ± 4.1% in the flexible electronic patch group, 44.2 ± 4.5% in the ES group, 49.7 ± 6.9% in the drug group, and 57.0 ± 2.7% in the control group. The remaining wound area percentage in the flexible electronic patch group was significantly reduced compared to the other three groups. By Day 10, the remaining wound areas were 6.6 ± 2.2% (flexible electronic patch group), 12.9 ± 5.1% (ES group), 18.6 ± 4.1% (drug group), and 36.9 ± 8.5% (control group), showing significant difference (*p* < 0.05, flexible electronic patch group versus ES group; *p* < 0.05, flexible electronic patch group versus control group). It demonstrated that the flexible electronic patch promotes wound healing (Figure 6B). Subsequently, rats were sacrificed, and skin tissue from the wound site was harvested for sectioning and staining. Hematoxylin and eosin (H&E) staining of wound tissue showed that the epidermal layer in the control group still exhibited defects and incomplete healing. The drug group displayed mild inflammation in the epidermis. In contrast, both the ES group and the flexible electronic patch group showed better healing outcomes (Figure 6C). These results indicate that both electrically controlled chlorhexidine release and electrical stimulation therapy can promote wound healing. Notably, the therapeutic effect of electrical stimulation was superior to that of chlorhexidine alone. The flexible electronic patch, by integrating the dual functions of electrical stimulation and controlled drug delivery, combines the advantages of both approaches, resulting in the most effective therapeutic outcome.

The wound healing mechanisms described can explain the therapeutic efficacy observed in this study. Healthy skin maintains a natural transepithelial potential (TEP). When injured, this potential generates an endogenous electric field that guides repair cells—including keratinocytes, fibroblasts, and macrophages—to the wound center via electro taxis. The applied ES can mimic and enhance this endogenous field [38, 39]. At the cellular level, ES alters the resting membrane potential, triggering targeted depolarization and opening voltage‐gated calcium and potassium channels. The resulting calcium influx acts as a secondary messenger, forcing key membrane receptors (such as EGFR and integrins) to cluster on the cathodic side of the cell. This establishment of cell polarity activates specific downstream kinase cascades: the PI3K/Akt pathway drives rapid actin polymerization for cell migration, while the MAPK/ERK pathway stimulates fibroblast proliferation and Type I collagen synthesis [38, 40, 41, 42]. Synergistically, these pathways upregulate VEGF expression, promoting angiogenesis to restore oxygen to hypoxic tissues [43, 44]. Beyond physical acceleration, ES actively modulates the immune response. It suppresses pro‐inflammatory cytokines (TNF‐α, IL‐6) and drives the phenotypic polarization of macrophages from the pro‐inflammatory M1 state to the regenerative M2 state [45, 46]. Finally, during the remodeling phase, electrical currents modulate the TGF‐β pathway to prevent excessive collagen deposition, reducing hypertrophic scarring [43, 47].

In our flexible electronic patch, this physical mechanism is synergistically paired with chemical intervention. While ES accelerates tissue reconstruction, the electro‐controlled release of chlorhexidine ensures elimination of bacterial infections, which is a primary cause of stalled healing and excessive inflammation. By eliminating this biological burden, the drug prevents infection‐related delays, allowing the electrical stimulation to function at maximum efficiency. Thus, dual functionality creates an optimal healing environment: the drug protects the wound, while the electrical field actively drives its closure.

### 2.7 Evaluation of the Tailorable Character of Flexible Electronic Patch in Wound Management

**FIGURE 7:**
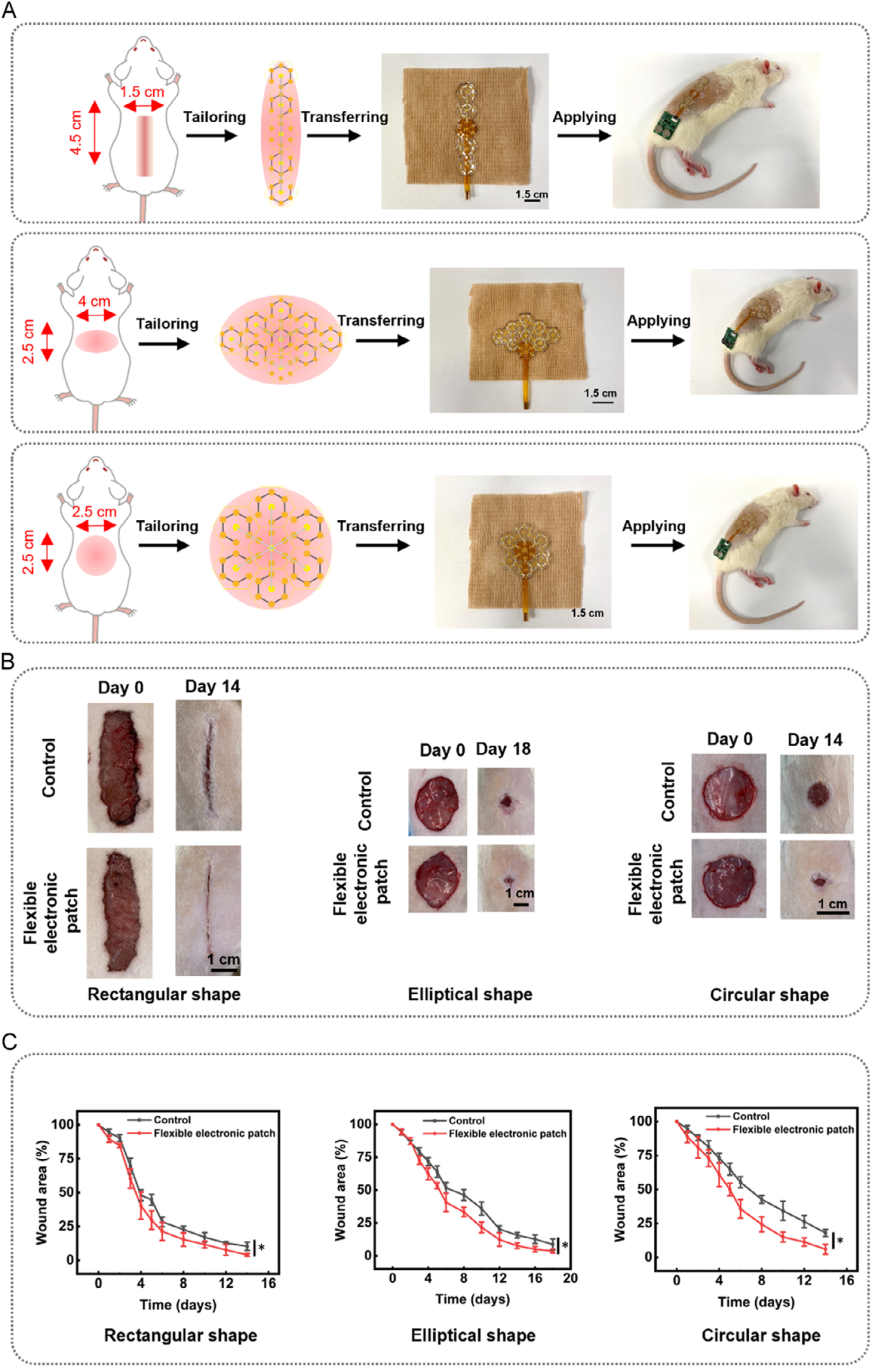
The therapeutic effect of the flexible electronic patch after being cut into different shapes. (A) Schematic diagram of the flexible electronic patch being cut into different shapes (rectangular shape, elliptical shape, circular shape) and transferred to a wound dressing for application on a rat; (B) Representative images of wounds; (C) The remaining wound area percentage. The data are presented as mean ± SD, *n* = 6. * represents *p* < 0.05.

To evaluate the tailorable character of the flexible electronic patch in wound management, we constructed three types of wound shapes in rat dorsal region (Figure 7, Figure S10, Figure S11, and Figure S12): a rectangular wound (4.5 cm × 1.5 cm), an elliptical wound (4 cm major axis, 2.5 cm minor axis), and a circular wound (2.5 cm diameter). Based on the shapes and dimensions of these wounds, the flexible electronic patches were cut into corresponding shapes and dimensions and transferred onto nonwoven fabrics for wound treatment. The rats with each wound model were divided into a control group and a treatment group (flexible electronic patch application). Over time, both the control and flexible electronic patch groups showed gradual wound healing, with the flexible electronic patch group demonstrating significantly faster healing rates. For rectangular wound, the remaining areas on Day 5 were 29.7 ± 6.8% (flexible electronic patch group) and 44.7 ± 4.0% (control group) of the initial area, and on Day 14, they were 4.1 ± 1.0% (flexible electronic patch group) and 10.3 ± 3.1% (control group). For elliptical wound, the remaining areas on Day 5 were 53.3 ± 2.3% (flexible electronic patch group) and 64.0 ± 4.4% (control group) of the initial area, and on Day 18, they were 3.7 ± 1.5% (flexible electronic patch group) and 8.7 ± 4.2% (control group). For circular wound, the remaining areas on Day 5 were 50.1 ± 4.6% (flexible electronic patch group) and 65.0 ± 4.4% (control group) of the initial area, and on Day 14, they were 6.3 ± 3.6% (flexible electronic patch group) and 18.2 ± 2.6% (control group). These results demonstrate that the flexible electronic patch can effectively adapt to wounds of different shapes and sizes, and that patches retain their electrical stimulation and electro‐controlled drug delivery performance after cutting, significantly promoting wound healing.

The realization of tailorable flexible electronic patch is made possible by the consistent functionality of the circuits, regardless of their size or shape. We designed a minimal unit incorporating electrical stimulation and electro‐responsive drug delivery capabilities. These units can be combined and expanded outward to achieve a tailorable structure. Here, a regular hexagon with full electrical functionality serves as the core minimum structural unit, extended to form a flexible electronic patch. Such minimal units are widely used in wearable electronics to improve detection sensitivity and control accuracy. For instance, Jung et al. developed a lightweight, flexible system that displays vibrotactile patterns via individual units or wireless networks, reducing size and weight while enhancing the haptic unit's strength and efficiency [48]. Similarly, Flavin et al. designed a miniaturized haptic unit that integrates with the skin to provide bistable, self‐sensing actuation capable of delivering dynamic and static stimuli through normal or shear forces [49].

Inspired by the structural design in these studies, we used a regular hexagon as the minimal structural unit to achieve tailorable performance. The hexagonal design shows clear advantages in wiring efficiency, signal transmission stability, and stress reduction. Among regular polygons, only equilateral triangles, squares, and regular hexagons can tessellate a plane. While tessellation with equilateral triangles requires rotation, which will complicate circuit design. For squares, the circuits have eight expansion directions outward, while regular hexagons provide six expansion directions, simplifying circuit design and improving system reliability. The equidistant spacing between adjacent hexagons allows uniform signal propagation, shortening the average communication distance between modules and reducing the overall application scope [50]. Furthermore, in flexible substrates subjected to dynamic mechanical deformation, sharp corners easily become stress concentration points. The obtuse angles of hexagons effectively reduce mechanical stress during substrate bending and stretching, thereby reducing the risk of microcracks and delamination in printed silver wires [51]. Accordingly, we designed a flexible electronic patch consisting of interconnected regular hexagonal units. Each unit contains seven conductive hydrogel discs: six placed near the edges and connected to the anode for drug delivery, and one at the center connected to the cathode for electrical stimulation to promote wound healing. When powered via the central unit, with conductive hydrogels interconnected by silver wires, the patch can be tailored to match the size and shape of the wound while keeping the central unit intact.

A few studies have explored the use of minimal structural units in tailorable electronic components, such as wireless power transfer sheets [52] and capacitive multi‐touch sensors [53]. However, wound management presents significantly more complex requirements. It demands rapid tailoring while maintaining advanced functionalities of electrical stimulation and electro‐controlled drug delivery—all within the challenging environment of a wound bed, which involves fluid exposure and dynamic changes in shape, size, and surface condition. Tailorable flexible electronic patches that satisfy these demanding conditions remain largely unexplored. Thus, the tailorable flexible electronic patch developed in this work offers a new solution for personalized wound treatment.

While this study demonstrates progress in developing tailorable and transferable flexible electronic patches, further improvements are still possible. A next step is the development of intelligent closed‐loop systems that adapt to the dynamic process of wound healing. Due to the unpredictable wound environments (e.g., variations in shape, size, exudate levels, tissue regeneration stages, and microbial activity), static, preprogramed electronic systems are often inadequate [54]. To achieve fully autonomous operation, wireless communication modules (e.g., NFC or Bluetooth) can be integrated into the patch. Because passive near‐field energy harvesting yields insufficient power for continuous high‐voltage electro‐therapies [55], this energy gap must be bridged using high‐output sources like flexible solid‐state lithium‐ion batteries [56, 57], ultrasound‐enhanced nanogenerators [58], or enzyme biofuel cells [59]. Meanwhile, wireless modules can be dedicated strictly to real‐time telemetry and therapeutic parameter control. Furthermore, arbitrary edge‐trimming typically serves conventional RF antennas and disables wireless functionality [60]. To preserve the patch's tailorable design, future iterations can adopt distributed fractal antenna layouts (such as Peano curves [61]) and H‐tree wiring topologies [52], which inherently maintain electromagnetic resonance even after peripheral segments are removed. Combining these redundant structures with self‐tuning RFID microchips to correct post‐cutting frequency shifts, while centralizing rigid components via an island‐bridge architecture [62], ensures robust wireless capabilities without compromising the dressing's customizable geometry. Future research would focus on creating an integrated sensing‐treatment‐feedback platform capable of real‐time adaptation [63]. This system would continuously monitor key wound parameters, such as fluid accumulation, inflammatory markers, and tissue conductivity [54]. Using embedded algorithms, the acquired data would dynamically modulate electrical parameters, including pulse frequency, amplitude, and spatial distribution, as well as drug release. By autonomously adjusting its functionality based on real‐time conditions, the dressing would minimize manual intervention and enhance healing efficiency through sustained optimal wound microenvironments.

## 3 Conclusions

This study developed a tailorable, transferable, flexible electronic patch for simultaneous electrical stimulation and electro‐controlled drug delivery in wound management. The patch overcomes limitations of prior flexible electronics by enabling customization to wounds of varying sizes through shape cutting. It can also be transferred onto commercial wound dressings, endowing them with dual functionality: electro‐controlled drug release and electrical stimulation. Animal studies validated the patch's wound‐healing efficacy and tailorability. The key innovation of this study is a new circuit connection method, which enables device tailoring, while a WTP technique imparts dual therapeutic functions to conventional dressings. This study provides a viable strategy for advancing flexible electronics research and clinical translation.

## 4 Experimental Section

### 4.1 Chemicals and Materials

Sodium chondroitin sulfate A, 1,4‐butanediol diglycidyl ether, sodium hydroxide, chlorhexidine acetate, methylene blue, cationic brilliant red 4g, brilliant blue, methyl orange, direct blue 15, carmine, PBS buffer solution, paraformaldehyde, PVA (Mw≈47,000), chitosan, acetic acid, and chloral hydrate were purchased from Aladdin Co., Ltd. (China). Conductive silver paste was purchased from OSBON Technology Co., Ltd. (China). WTP paper was purchased from Winner Transfer (China). The PU film was obtained from Qingdao Haishi Hainuo Group (China). It is classified as a Class I medical device, about 10 micrometers thick, and possesses excellent waterproof and flexible properties. To ensure standardization, all clinical wound dressings used in this study (including cotton gauze, nonwoven fabrics, medical plaster, silicone foam dressings, and hydrogel dressings) were commercially purchased from Qingdao Haishi Hainuo Group (China) and used as received without further modification.

### 4.2 Preparation of Chondroitin Sulfate Hydrogel

First, 1 g of CS powder was dissolved in 3.76 mL of 1 M NaOH solution at room temperature (25°C). Meanwhile, 890 μL of 1,4‐butanediol diglycidyl ether was added to the solution, and the mixture was stirred at 1 revolution per second for 5 min to yield a yellow hydrogel precursor solution. Subsequently, the corresponding volume of the hydrogel precursor solution was added to the solution. Then, the hydrogel precursor solution was aliquoted into drops (100 μL per drop). The hydrogel precursor solution was heated in a 60°C oven (101‐3A, Kaile Instruments Co., Ltd. (China)) for 1 h to obtain a brown dried hydrogel network. Finally, the brown dried hydrogel network was immersed in PBS buffer for 24 h to reach swelling equilibrium (the PBS buffer was changed every 6 h) to obtain a swollen CS hydrogel (Figure S1).

### 4.3 Characterizations of Chondroitin Sulfate Hydrogel

#### 4.3.1 Swelling Ratio

The initial weight of the dried hydrogel was immersed in PBS, and its weight was measured every 5 min. The swelling ratio was calculated by dividing the weight after water absorption by the initial weight before absorption.

#### 4.3.2 Drug‐Loading Rate

The hydrogel was immersed in 10 mL of 0.1% (w/w) chlorhexidine acetate solution. Every 1 h, 100 μL of the solution was withdrawn, and the absorbance of the solution was measured at 290 nm using a microplate reader. The remaining concentration of chlorhexidine acetate in the solution was calculated based on the absorbance. The amount of chlorhexidine acetate reduced compared to the initial solution corresponds to the drugs loaded by the hydrogel. The drug‐loading rate was determined by dividing the mass of the drug loaded into the hydrogel by the mass of the hydrogel before drug loading.

#### 4.3.3 Drug Release Rate

The drug‐loaded hydrogels were immersed in 10 mL of PBS, and square‐wave voltage signals of 1 V, 2 V, and 3 V (at a frequency of 50 Hz) were applied. For the spontaneous release experiments (control), the hydrogels were not connected to a power source. 100 μL of the solution was withdrawn every 30 min, and the absorbance of the solution was measured at 290 nm using a microplate reader to calculate the concentration of chlorhexidine acetate in the solution. The drug release rate was determined by dividing the mass of the drug released by the mass of the drug loaded into the hydrogel.

### 4.4 Fabrication of the Flexible Circuit Board

The TPS7A2028 linear regulator ensures a stable + 2.8 V output, while the TPS60403 DC–DC converter generates a −2.8 V output within its input range of + 1.6 V to + 5.5 V.

The LMC555 timer is used as an oscillator to output continuous rectangular pulse voltage at a specified frequency. The oscillation frequency is:

$$f=\frac{1.44}{\left(R_{1}+2R_{2}\right)C_{3}}$$

While R1 is 576 kΩ, R2 is 1.15 MΩ and C3 is 10 nF, resulting in an oscillation frequency of ≈ 50 Hz. The SN74LVC2G17 was used to decrease the influence of the next device. The OPA330 is a low‐voltage dual operational amplifier with two output channels. One operational amplifier is configured as an inverting amplifier (+INB, ‐INB, and OUTB), generating a negative pulse voltage at OUTB for electrical stimulation of wound healing. The other operational amplifier is configured as a voltage follower, producing a positive rectangular pulse voltage at OUTA for electro‐controlled drug delivery. Finally, the flexible circuit board was positioned between the anode and cathode layers, completing the flexible electronic patch fabrication. The electrical conductivity of the flexible electronic patch was characterized by measuring voltage waveforms using an oscilloscope (UNI‐T UPO1202S‐E) with a sampling period of 10^−5^ s.

### 4.5 Fabrication of the Flexible Electronic Patch

The flexible electronic patch was fabricated through followed process: preparation of the anode layer, fabrication of the holes, assembly of the CS hydrogels, and preparation of the cathode layer.

#### 4.5.1 Preparation of the Anode Layer

The anode circuit was fabricated on WTP paper, with holes punched in the designated areas of WTP paper for hydrogel placement. Subsequently, conductive silver paste was screen‐printed onto the WTP paper using a predesigned circuit stencil. The stencil specifications included a mesh count of 200–300, with pore diameters of 0.054–0.077 mm and wire diameters of 0.03–0.05 mm. The designed line width of the stencil was 0.5 mm, resulting in a printed conductive line width of ≈ 1 mm. The printed layer was then cured at 80°C for 1 h.

#### 4.5.2 Fabrication of the Holes

To prevent short circuits between these wires, we print them separately on the WTP paper and the PU film. To ensure the hydrogel contacts the skin and the negative electrode wire contacts the hydrogel used for electrical stimulation therapy in the center of each hexagon, we created holes in the WTP and the PU film. The holes in the patch were created using a punching machine on the WTP and the PU film, with a diameter of 1.5mm. As shown in Figure S5, the dark yellow circles represent the distributions of the holes where the CS hydrogel connecting to the positive electrode is placed, and the light yellow circles represent the distributions of the holes where the CS hydrogel connecting to the negative electrode is placed.

#### 4.5.3 Hydrogel Assembly

CS hydrogel was first fabricated into circular discs with a diameter of 2 mm and a thickness of 1 mm. The hydrogel discs were immersed in 10 mL of 0.1% (w/w) chlorhexidine acetate solution for 24 h to load the drugs. After freeze‐drying, CS hydrogel discs were then assembled into the prepunched holes.

#### 4.5.4 Preparation of the Cathode Layer

The cathode layer was fabricated using a PU waterproof film. Conductive silver paste was screen‐printed onto the PU film using a stencil. The stencil specifications included a mesh count of 200–300, with pore diameters of 0.054–0.077 mm and wire diameters of 0.03–0.05 mm. The designed line width of the stencil was 0.5 mm, resulting in a printed conductive line width of ≈ 1 mm. The printed layer was then cured at 80°C for 1 h.

#### 4.5.5 Assembly of Flexible Patches

First, place the anode layer, then place the CS hydrogel in the prepunched holes, followed by the installation of the flexible circuit board, then the installation of the PU waterproof film to prevent short circuits, and finally the installation of the cathode layer (Figure S6).

#### 4.5.6 Freeze‐Drying

Freeze‐drying pretreatment is required before SEM imaging. First, the prepared hydrogels are placed in a ‐80°C refrigerator for 12 h to ensure complete freezing of the internal water. Subsequently, the frozen samples are transferred into a vacuum freeze‐dryer (Alpha 2–4 LSCbasic, Martin Christ Gefriertrocknungsanlagen (Germany)) for 72 h to remove water via ice sublimation, yielding the final dried samples.

### 4.6 Water‐Transfer Printing of the Patch

#### 4.6.1 Water‐Transfer Printing Process

Clinically used and widely studied wound dressings (including cotton gauze, nonwoven fabrics, plaster, silicone foam dressings, hydrogel dressings, and electrospinning dressing) were selected as examples to evaluate the WTP capability of the flexible electronic patch. First, the plastic protective film on the back of the prepared flexible electronic patch was peeled off, and the patch was adhered to the wound dressing. Sterile water was applied to the WTP paper to moisten it, followed by a 30‐second waiting period. The WTP paper was then carefully removed, transferring the flexible electronic patch onto the wound dressing.

#### 4.6.2 Water‐Transfer Printing Success Rate

Six types of wound dressings were selected for this experiment. Premade patches were applied to the wound dressings for WTP. The success rate of WTP was calculated by comparing the theoretical transfer area with the actual transfer area. Each experiment was repeated three times, and the average value was calculated.

### 4.7 Water Transmission Rate and Water Vapor Transmission Rate Test

#### 4.7.1 Water Transmission Rate Test

The WTR of the dressing was assessed using a static permeation apparatus. The water‐transfer printed dressing sample was horizontally fixed between the upper and lower chambers of a diffusion chamber. 10 mL of deionized water was added to the upper chamber, and 5 mL of 1% chlorhexidine solution (not in contact with the dressing) was added to the lower chamber. All samples were incubated in a constant temperature and humidity chamber at 37°C and 50 ± 5% relative humidity (RH) for 1 h. Because directly measuring the volume of water has a large error, we accurately determine the volume of the liquid by measuring the absorbance value of the chlorhexidine solution. After 1 h, the absorbance of the chlorhexidine solution in the lower chamber was measured, and the amount of deionized water that permeated into the dressing sample was calculated. All measurements were repeated three times, and results are expressed as mean ± standard deviation (SD).

#### 4.7.2 Water Vapor Transmission Rate Test

The WVTR was determined using ASTM E96. This indicator reflects the air permeability of wound dressings. ASTM E96 is a standard test method developed by the ASTM International organization for determining the WVTR of various materials (including permeable and semi‐permeable materials, such as wound dressings) and providing reliable WVTR data to assess the air permeability of dressings. The dressing sample was securely placed between the upper and lower chambers of the diffusion chamber. 20 mL of deionized water was added to the lower chamber, and the sampling port of the lower chamber was sealed. The upper chamber was exposed to air. The initial weight of the recording device (excluding the dressing sample) was m_0_. All samples were incubated in a constant temperature and humidity chamber at 37°C and 50 ± 5% RH for 24 h. After incubation, the final weight of the recording device (excluding the dressing sample) was m_t_. WVTR was calculated using the following formula:

$$\text{WVTR}=\frac{m_{0}-m_{t}}{A\times t}\times 100\%$$

where *A* is the effective test area of the sample (m^2^) and *t* is the test time (h). All measurements were repeated three times, and results are expressed as mean ± SD.

### 4.8 Evaluation of the Therapeutic Efficacy of the Flexible Electronic Patch in Wound Healing In Vivo

All animal experiments were approved by the Institutional Animal Care Committee of Guangxi University (Reference Number: GXU‐2024−207). Sprague‐Dawley male rats (6 weeks old, weighting 200 ± 20 g) were purchased from Guangxi Medical University. Rats were randomly divided into four groups: no treatment group (control), the only drug group, the only electrostimulation group (only ES), and the combination group (flexible electronic patch), with six rats in each group. The rats were anesthetized with chloral hydrate (7% w/v, 0.7 mL per 100 g). A circular wound with a diameter of 1.5 cm was created on the right dorsal region of each rat. To eliminate potential interference from commercial wound dressings with varying compositions and properties during animal experiments, cotton spinning was employed as the uniform dressing substrate across all experimental groups. The dressings applied in the treatment groups were prepared by transferring the flexible electronic patch onto cotton spinning substrates. The control group received only pure cotton spinning dressings to cover the wound sites. Three treatment groups utilized cotton spinning dressings integrated with water‐transfer printed flexible patches. After preparation and prior to animal experiments, the patches were placed in a clean bench and subjected to ultraviolet sterilization for 30 min to ensure their sterility and avoid interference with the animal experiments. Subsequently, the experimental groups were treated daily for 1 hr with drug‐only therapy using an electro‐controlled drug delivery flexible electronic patch without electrostimulation, electrostimulation‐only therapy using no drug‐loaded flexible electronic patch, or combination therapy (drug + electrostimulation), respectively. The wound area of all rats, including the control group, was recorded. After 10 days, the rats were sacrificed, and skin tissue was collected from the wound site for paraffin embedding and processed into histological sections. The skin sections were stained with H&E and observed under a microscope to analyze the tissue structure.

### 4.9 Evaluation of the Tailorable Character of Flexible Electronic Patch in Wound Management

Sprague‐Dawley male rats (six weeks old, weighing 200 ± 20 g) were randomly divided into three groups (12 rats per group). Rectangular (4 cm × 1.5 cm), elliptical (4 cm × 2.5 cm), and circular (diameter: 2.5 cm) wounds were created separately on the dorsal regions of these rats (Each group was created with one type of wound). Each wound model was further subdivided into two subgroups: the control group (no treatment) and the flexible electronic patch treatment group, with six rats per subgroup. The flexible electronic patches were custom‐cut according to the wound size and shape, ensuring the central interface part of the patch was retained. Subsequently, the tailored patches were transferred onto commercial wound dressings and applied to the wounds of experimental subgroup rats for treatment for 1 h every day. The wound area of all rats, including the control group, was recorded.

### 4.10 Statistical Analysis

In this work, all of the data were presented as the mean ± SD (mean ± SD) from three independent in vitro experiments and six independent in vivo experiments. Statistical comparisons were performed using a two‐tailed, paired Student's T test, with a *p*‐value of less than 0.05 deemed statistically significant. OriginPro (2025) was used for statistical analysis and drawing statistical figures. ns represents *p* > 0.05, * represents *p* < 0.05.

## Supporting Information

Additional supporting information can be found online in the Supporting Information section.

## Author Contributions

**Rongyan He**: writing – review & editing, resources, project administration, methodology, investigation, funding acquisition, conceptualization. **Qiuyu Cao**: writing – review & editing, writing – original draft, visualization, resources, methodology, investigation, formal analysis, data curation, conceptualization. **Wenhui Yan, Shuting Xiao, Xiaoying Liang**: data curation. **Yuxiu Ye**: investigation. **Dongting Zhangsun**: supervision, conceptualization. **Sulan Luo**: writing – review & editing, resources, project administration, funding acquisition, conceptualization.

## Funding

This research was funded in part by ‘Dai Tu Yi Zhi’ Project of Key R & D Program of Guangxi (GUIKE AA25069003), Guangxi Natural Science Foundation (2025GXNSFBA069577), Major International Joint Research Project of National Natural Science Foundation of China (82320108019), the National Natural Science Foundation of China (No. 42376112 and No. 82360698), Key Project of Natural Science Foundation of Guangxi (No. 2024GXNSFDA999003), Major Intergovernmental Joint Research Project of National Key R & D Program of China (2022YFE0132700), Guangxi Qingmiao Talent Support Program.

## Conflicts of Interest

The authors declare no conflicts of interest.

## Clinical Trial Number

Not applicable. This study does not involve clinical trials.

## Supporting information

Supplementary Material

## Data Availability

The data that support the findings of this study are available from the corresponding author upon reasonable request.
