# Supplementary material for: A Tailorable and Transferable Flexible Patch for Simultaneous Electrostimulation and Electro‐Controlled Drug Delivery in Wound Management
Source: Small Sci. 2026 Apr 21;6(4):e202500570. doi: 10.1002/smsc.202500570 (PMC13099368; doi:10.1002/smsc.202500570)
Supplement: Supplementary file 1 — Supplementary Material [file SMSC-6-e202500570-s001.zip › Revised_Supporting Information.pdf]

Supporting Information

A tailorable and transferable flexible patch for simultaneous electrostimulation and electro-controlled drug delivery in wound management

Rongyan He#, Qiuyu Cao#, Wenhui Yan, Shuting Xiao, Xiaoying Liang, Yuxiu Ye, DongtingZhangsun, Sulan Luo*

Guangxi Key Laboratory of Special Biomedicine, School of Medicine, Guangxi University, Nanning, 530004, China.

# Authors contributed equally.

* Corresponding author: sulan2021@gxu.edu.cn.

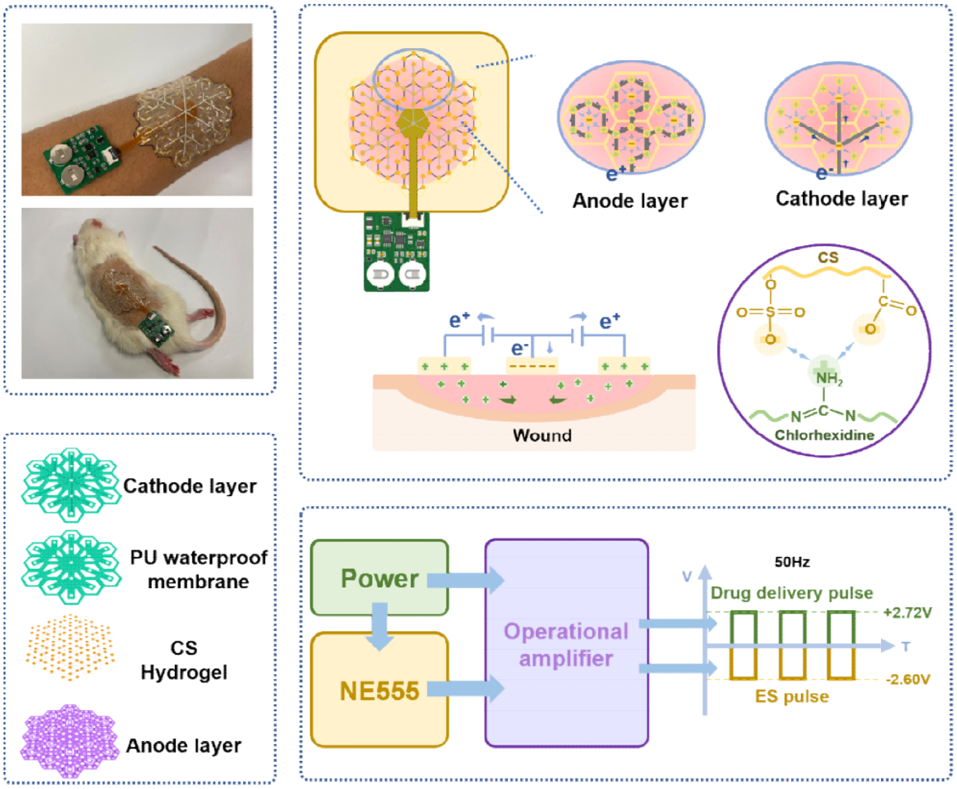

Graphical abstract

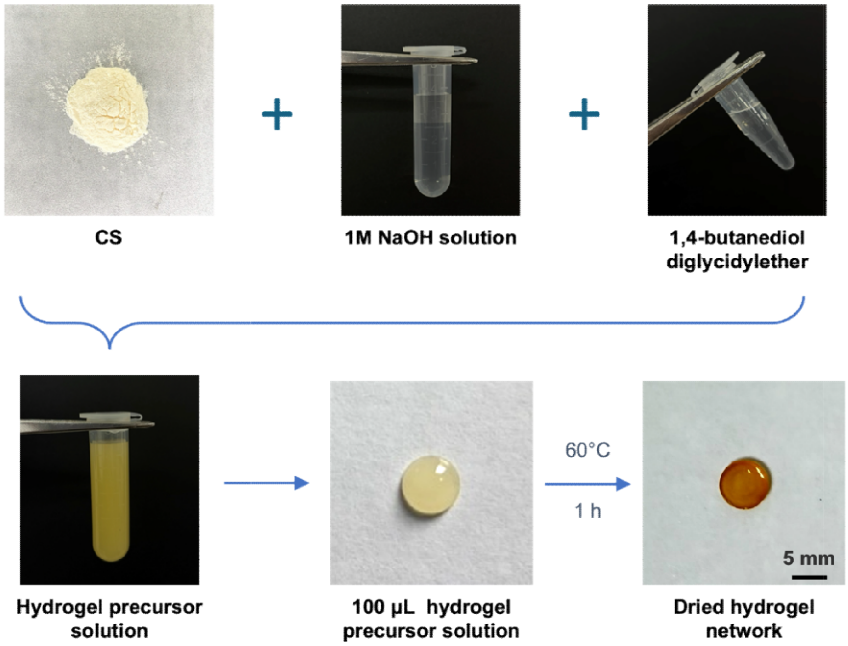

Figure S1. The preparation of CS hydrogel. CS powder is dissolved in NaOH and mixed with 1,4-butanediol diglycidyl ether to form a yellow precursor solution. The solution is aliquoted into drops and thermally cross-linked at 60 °C for 1 h, resulting in a brown dried hydrogel network.

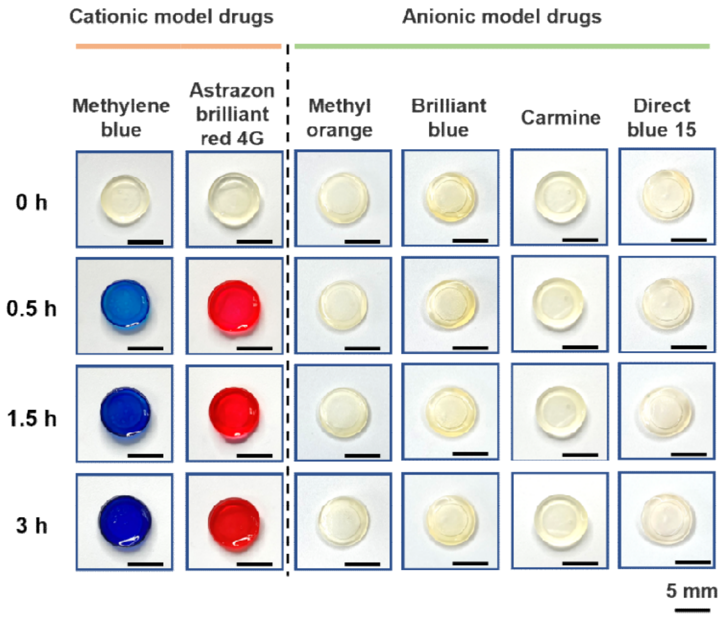

Figure S2. The preparation and cationic drugs affinity of CS hydrogel. Methylene blue and astrazon brilliant red 4G as model cationic drugs while methyl orange, brilliant blue, carmine, and direct blue 15 as control.

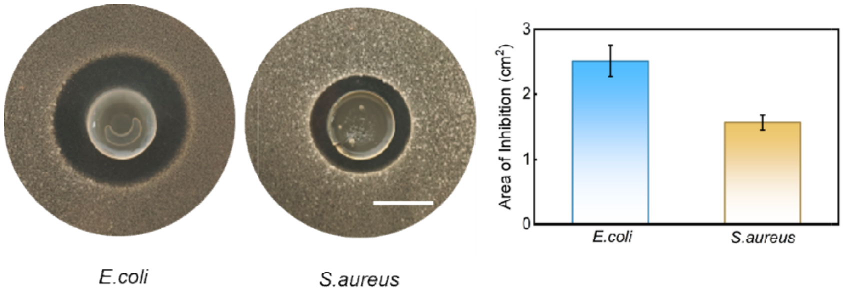

Figure S3. The antibacterial activity of hydrogel against *E. coli* and *S. aureus*. Scale bar = 1 cm.The data are presented as mean ± SD, n = 3.

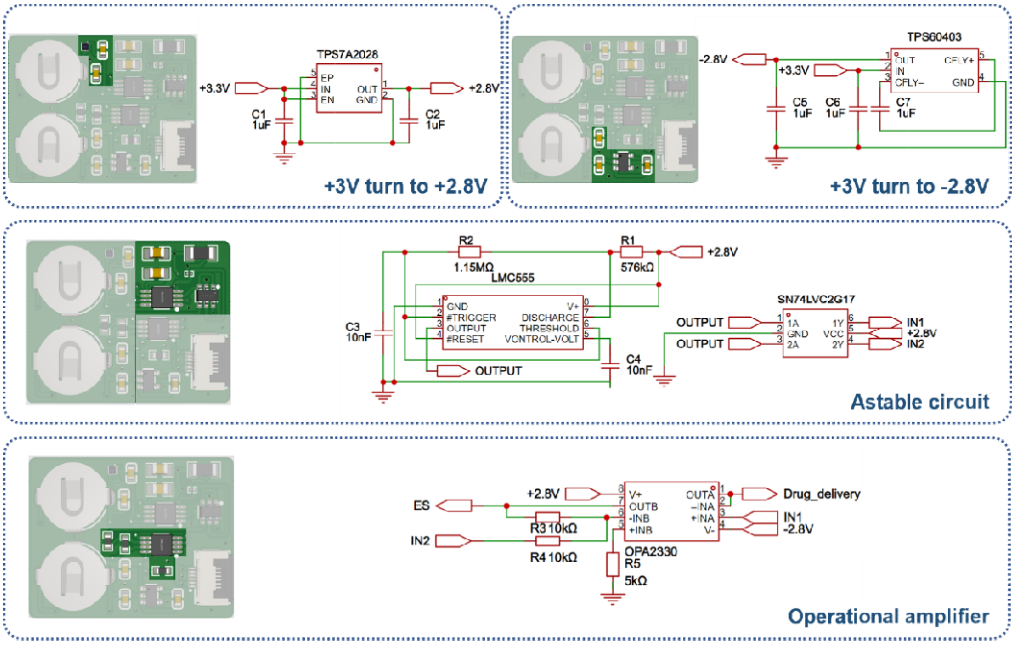

Figure S4. Circuit diagram of the miniaturized circuit board.

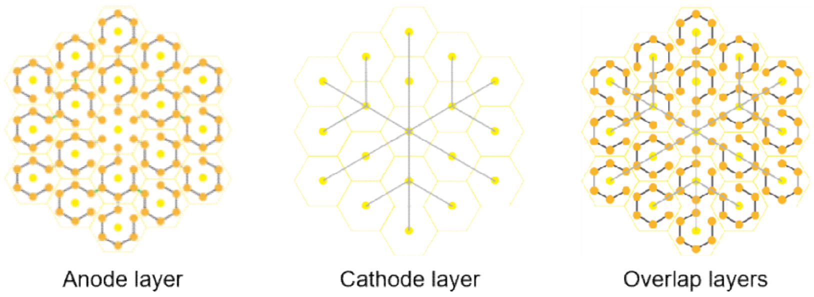

Figure S5. Schematic diagram of hydrogels, silver wires distribution.

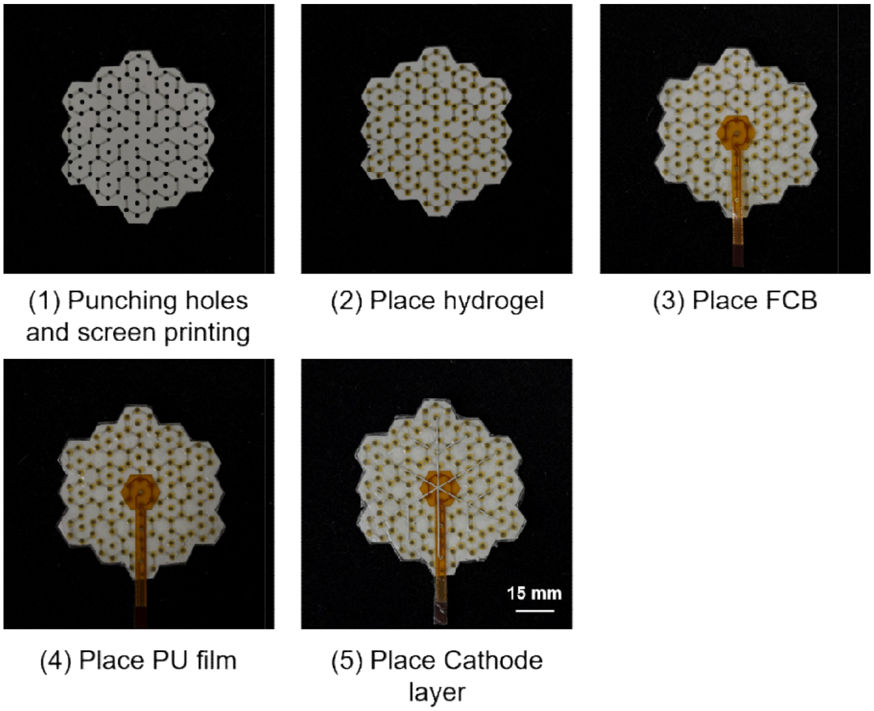

Figure S6. Fabrication of the flexible electronic patch. The sequential fabrication process involves: (1) preparing the anode layer by screen-printing conductive silver paste onto a pre-punched WTP paper substrate; (2) embedding the drug-loaded CS hydrogel discs into the designated perforations; (3) integrating the FPC to the central module; (4) applying a pre-punched PU waterproof film to serve as an insulating layer between the electrodes; and (5) completing the device by laminating the cathode layer, which consists of a second PU film screen-printed with silver conductive tracks.

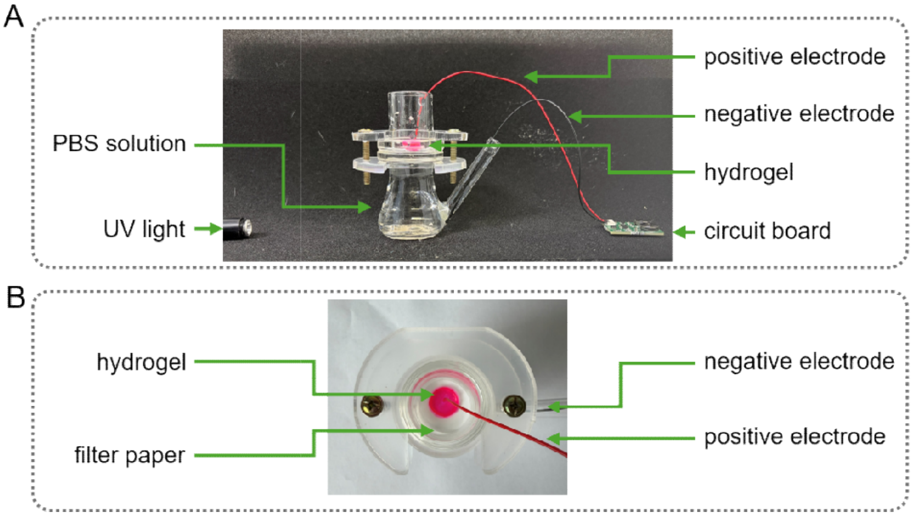

Figure S7. The experimental setup of drug release from CS hydrogels. (A) Front view of the experimental setup; (B) Top view of the experimental setup.

A

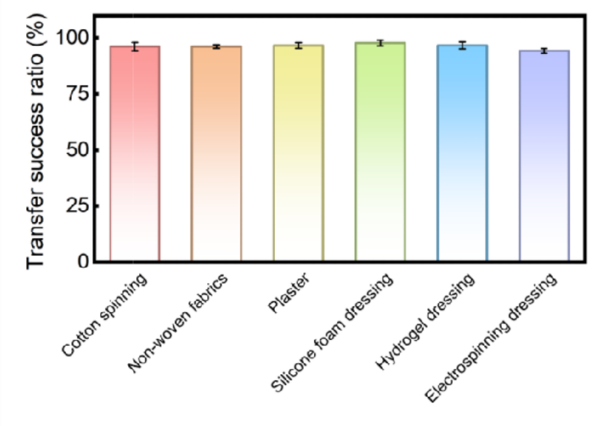

B

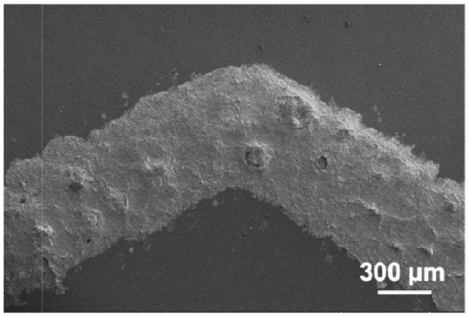

C

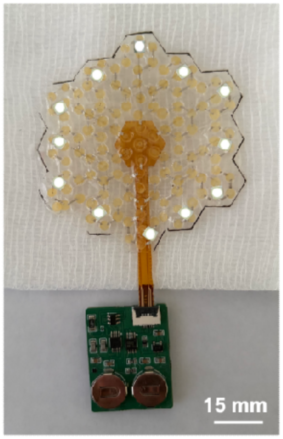

Figure S8. Performance evaluation after water transfer printing.(A) The transfer success ratio on different dressings; (B) The SEM images of silver wire on substrate materials; (C) LEDs on the patch.The data are presented as mean ± SD, n = 3.

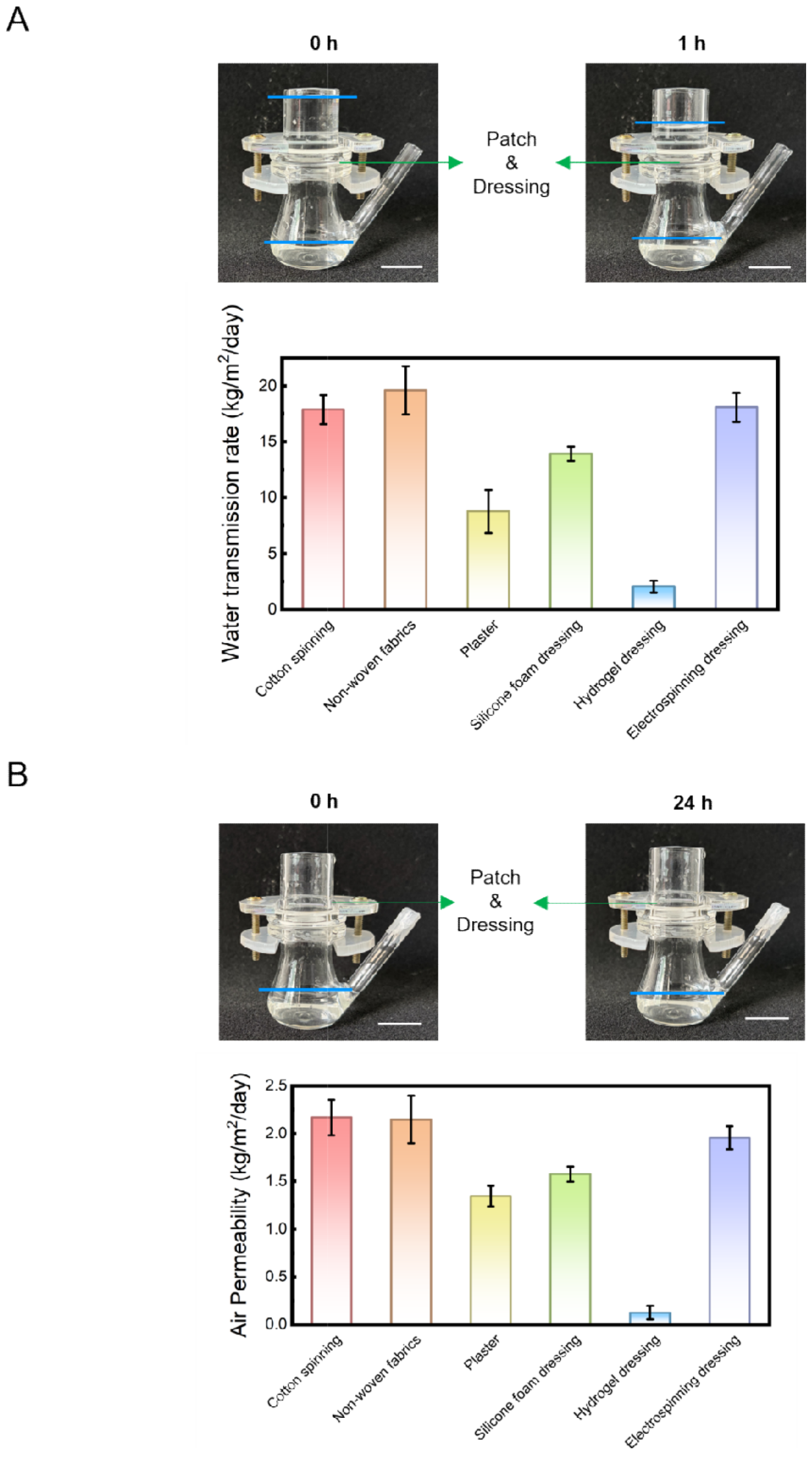

Figure S9. WTR and WVTR test of the patch. (A) The experimental setup photos and statistical charts of the WTR test; (B) The experimental setup photos and statistical charts of the WVTR test. The blue horizontal line represents the liquid level.Scale bar = 3 cm.The data are presented as mean ± SD, n = 3.

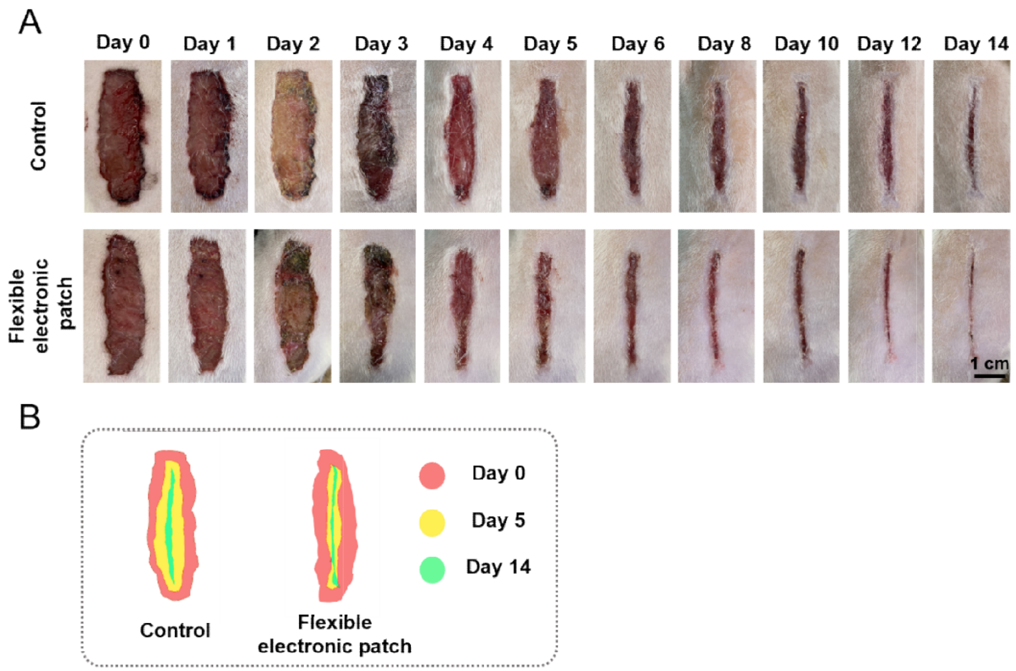

Figure S10. The therapeutic effect of the flexible electronic patch after being cut into a rectangular shape. (A) Representative images of wounds; (B) Traces of wound-bed closure in different treatment groups.

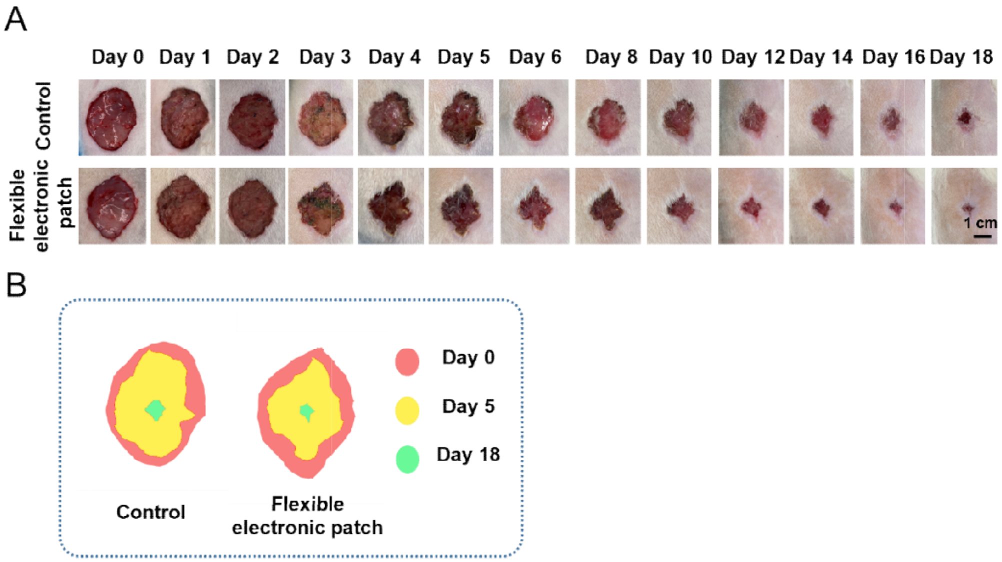

Figure S11. The therapeutic effect of the flexible electronic patch after being cut into an elliptical shape. (A) Representative images of wounds; (B) Traces of wound-bed closure in different treatment groups.

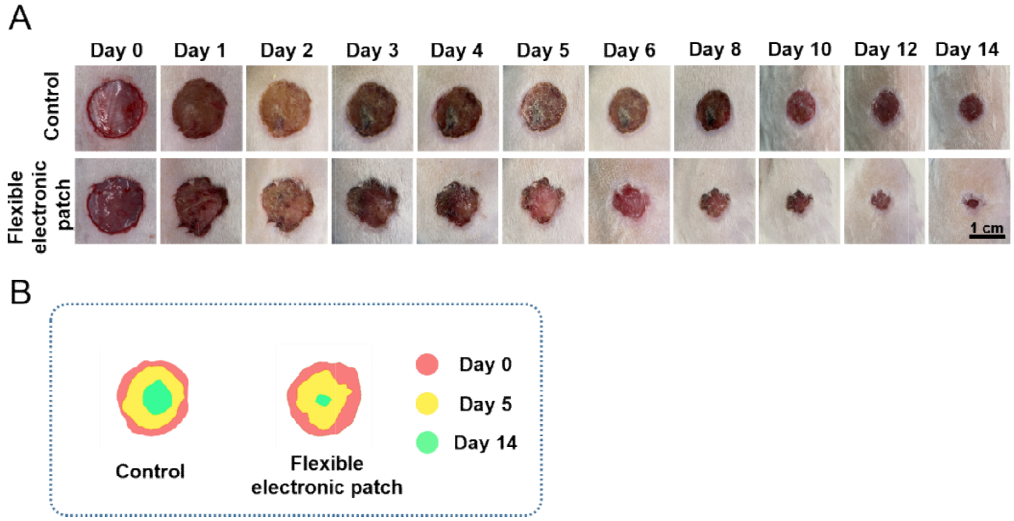

Figure S12. The therapeutic effect of the flexible electronic patch after being cut into a circular shape. (A) Representative images of wounds; (B) Traces of wound-bed closure in different treatment groups.

Video 1. The transfer process of the flexible electronic patch onto a commercial wound dressing using water-transfer printing method.

Video 2. Rats fitted with a flexible electronic patch for therapeutic intervention.
